# Ethnicity, sex, FADS genetic variation, and hormonal contraceptive use influence delta-5- and delta-6-desaturase indices and plasma docosahexaenoic acid concentration in young Canadian adults: a cross-sectional study

**DOI:** 10.1186/s12986-015-0010-9

**Published:** 2015-04-21

**Authors:** Salma A Abdelmagid, Shannon E Clarke, Kaitlin Roke, Daiva E Nielsen, Alaa Badawi, Ahmed El-Sohemy, David M Mutch, David WL Ma

**Affiliations:** Department of Human Health and Nutritional Sciences, College of Biological Science, University of Guelph, Animal Science/Nutrition Building, 491 Gordon Street, Guelph, ON N1G 2W1 Canada; Department of Nutritional Sciences, University of Toronto, Toronto, ON Canada; Office for Biotechnology, Genomics and Population Health, Public Health Agency of Canada, Toronto, ON Canada

## Abstract

**Background:**

There is great interest in the relationship between polyunsaturated fatty acids and health. Yet, the combinatory effect of factors such as sex, ethnicity, genetic polymorphisms and hormonal contraceptives (HC) on the concentrations of these fatty acids is unknown. Therefore, we sought to determine the effects of *FADS* polymorphisms, and HC use in females, on aggregate desaturase indices (ADI), and plasma docosahexaenoic acid (DHA) concentrations in Caucasian and East Asian males and females.

**Methods:**

Fasting plasma samples were collected from subjects (Caucasian males: 113 and females: 298; East Asian males: 98 and females: 277) from the Toronto Nutrigenomics and Health Study. Fatty acid concentrations were measured by gas chromatography. ADI were estimated by dividing concentrations of arachidonic acid by linoleic acid (n-6 ADI) and eicosapentaenoic acid (EPA) by α-linolenic acid (n-3 ADI). [DHA/EPA] desaturase index was used to determine effects of *FADS2* polymorphisms and HC use on EPA conversion to DHA.

**Results:**

In Caucasians, associations between n-6 ADI and multiple SNP (*FADS1* rs174547, FADS2 rs174576, and rs174611 in males; *FADS1* rs174547, *FADS2* rs174570, rs174576, rs174679, rs174611, rs174593, rs174626, rs2072114, rs2845573, and rs2851682 in females) withstood multiple testing. In East Asian females, 5 SNP-n-6 ADI associations (*FADS2* rs174602, rs174626, rs2072114, rs2845573, and rs2851682) withstood multiple testing. One *FADS2* SNP was associated with altered [DHA/EPA] desaturase index in Caucasian females only (rs174576, p < 0.0001). HC use had a significant effect on DHA concentrations in Caucasian females only (P < 0.0001).

**Conclusions:**

We demonstrate ethnic- and sex-specific effects of *FADS* polymorphisms on desaturase indices, and ethnic-specific effect of HC use on plasma DHA concentrations.

**Electronic supplementary material:**

The online version of this article (doi:10.1186/s12986-015-0010-9) contains supplementary material, which is available to authorized users.

## Background

Fatty acid desaturase 1 (*FADS1*) and fatty acid desaturase 2 (*FADS2*) encode the enzymes delta-5-desaturase (D5D) and delta-6-desaturase (D6D), respectively [1-5]. D5D and D6D are essential enzymes in the conversion of n-6 and n-3 polyunsaturated fatty acids (PUFA) to their longer chain products. Through a series of desaturation and elongation events, the n-6 PUFA linoleic acid (LA) is converted to arachidonic acid (AA) while the n-3 PUFA α-linolenic acid (ALA) is converted to eicosapentaenoic acid (EPA) and docosahexaenoic acid (DHA).

In recent years, a large body of work has addressed the effects of n-3 and n-6 PUFA on different aspects of our health (reviewed in [6]). Many of the reported biological effects of n-3 and n-6 PUFA seem to be mediated primarily by AA, EPA and DHA [6]. AA is a precursor of both anti-inflammatory, pro-inflammatory, pro-atherogenic and prothrombotic mediators, while EPA and DHA have been shown to play a role in cognitive development and in protection against cardiovascular disease and inflammatory conditions [7-10].

Several factors have been implicated in the alteration of endogenous fatty acid levels. These include diet, genetic variations and hormonal regulation [11,12]. Single nucleotide polymorphisms (SNP) in *FADS1* and *FADS2* have been shown to alter circulating FA levels reflected in changes in corresponding estimates of D5D and D6D enzymatic activity [13-15]. Moreover, *FADS* genetic variations have been shown to be associated with increased HOMA-IR and insulin resistance as well as hsCRP levels; thus, *FADS* polymorphisms can play a role in modifying disease risk [16,17]. In addition, another modifier is hormonal contraceptives (HC) which have been positively correlated with DHA levels in women [18,19].

We have previously shown the differential influence of *FADS* polymorphisms on D5D and D6D desaturation indices depending on ethnicity (Caucasian *vs.* East Asian) [20]; however, differential effects of *FADS* genetic variations based on sex are yet to be determined. In addition, the effects of hormonal contraceptive use on endogenous DHA concentrations in different ethnicities are also unknown. Identification of factors that differentially influence circulating fatty acid concentrations will advance our understanding of sex- and ethnic-specific aspects of fatty acid metabolism. Thus, in this study we sought to determine the differential effects of *FADS* polymorphisms and HC use on desaturase indices and circulating DHA concentrations in Caucasian and East Asian males and females.

## Subjects and methods

### Study population

Participants (total n = 786; Caucasians: males = 113, females = 298; East Asians: males = 98, females = 277) between the ages of 20–29 were recruited as part of the cross-sectional Toronto Nutrigenomics and Health (TNH) Study [21] between September 2004 and July 2009. Written informed consent was obtained from all participants. Subjects were free-living and were required to fast overnight for a minimum of 12 h prior to collection of blood. Standard clinical procedure was followed for the measurement of glucose, insulin, total-, and HDL-cholesterol, triglycerides, and free fatty acids [21]. HOMA-IR was calculated using the homeostasis model assessment method [22]. For the purpose of fatty acid analysis, plasma was separated from blood samples by centrifugation. Plasma samples were then frozen and stored at −80°C. Anthropometric measurements were recorded for all participants and health, life style, and food frequency questionnaires were completed by subjects. Completed questionnaires were used to calculate total energy intake from fat and physical activity scores, as previously described [21,23]. Ethnicity (Caucasians and East Asians) was the only inclusion criterion. Subjects were not excluded if they were fish or supplement consumers; however, to address dietary omega-3 and omega-6 intake regression models were adjusted for intake levels of LA, ALA, EPA and DHA obtained from FFQ*.* Women who were pregnant or breastfeeding were not included in the study. No other exclusion criteria were included in these analyses. The study protocol was approved by the Research Ethics Boards at the University of Toronto and University of Guelph.

### Gas chromatography analysis

Plasma total lipids were extracted and analyzed as described previously [20,24]. Free fatty acid C17:0 was used as an internal standard (5 μg of 1 mg/ml stock) and was used to calculate fatty acid concentrations (μg/ml). N-3 and n-6 aggregate desaturase indices (ADI) were calculated by dividing concentrations of EPA by ALA and AA by LA, respectively [20]. [DHA/ALA] and [DHA/EPA] desaturase indices were calculated by dividing concentrations of DHA by ALA and DHA by EPA, respectively. These indices were used to investigate the effects of *FADS1/2* polymorphisms and HC use on conversion of ALA to DHA and EPA to DHA, respectively.

### Genotyping

Identification of *FADS1*/*FADS2* SNP, selection of SNP and genotyping was performed as described previously [20]. Briefly, SNP in *FADS1* and *FADS2* were identified independently with the International HapMap Project SNP database. Tag SNP (tSNP) were selected with a minor allele frequency (MAF) greater than 0.05 and pairwise tagging (*r*^2^ ≥ 0.8), leading to the identification of 19 tSNP in total (3 in *FADS1* and 16 in *FADS2* that captured more than 43 initially identified SNP). Linkage disequilibrium (LD) was examined between the 19 tSNP identified using the SNP Annotation and Proxy Search (SNAP) database. Two tSNP were in high LD (*r*^2^ ≥ 0.8): rs174547 in *FADS1* and rs174576 in *FADS2*. Sequenom MassARRAY platform was used for genotyping, which is based on detection through MALDI-TOF MS (Mass Array, Sequenom, San Diego, CA). All 19 tSNP were replicated in 29 randomly selected DNA samples and 100% concordance was achieved [17].

### Statistical analysis of data

Results are expressed as mean ± standard error mean (SEM). Data analysis and testing for Hardy-Weinberg equilibrium was carried out using JMP genomics software V5 (SAS Institute, Cary, NC). A student’s t-test was used to determine differences in fatty acid concentrations and ratios between males and females. A Tukey’s Honestly Significant Difference post-hoc test was used to determine differences in desaturase indices for each genotype. P-values of analyses of fatty acid levels or desaturase indices were determined using multiple linear regression models which were adjusted for BMI, age, dietary LA, ALA, EPA and DHA, % total energy from dietary fat and physical activity. Multiple linear regression models were used to identify associations between individual desaturase indices and SNP or HC use and were adjusted for BMI, age, dietary LA, ALA, EPA and DHA, % total energy from dietary fat and physical activity. Due to the high LD known to exist between SNP in the *FADS* gene cluster, the SNP with the strongest association (i.e., smallest p-value) was subsequently included as a covariate in repeat linear regression models of identified SNP-ADI associations to determine whether these associations were dependent or independent of the covariate SNP. If the originally identified association was lost after addition of the covariate SNP to the model, the effect of the SNP investigated was considered dependent on covariate SNP. If the association remained significant, the effect of the SNP investigated was considered to be independent of the covariate SNP. A p-value of < 0.05 was considered statistically significant. To determine p-value cut off for multiple testing, 0.05 was divided by the number of total linear regression analyses performed for associations of SNP and desaturase indices (160 analyses) which resulted in a significant p-value cut off of 0.0003.

## Results

### Study population

Within Caucasian males and females, there were no significant differences in BMI, energy intake from fats or levels of triglycerides and free fatty acids. However, females had significantly higher values of HOMA-IR and higher levels of insulin, total cholesterol, HDL-cholesterol and LDL-cholesterol and significantly lower levels of glucose compared to males (Table 1). In East Asian males and females, there were no significant differences in values of HOMA-IR and energy intake from fats or levels of insulin, LDL-cholesterol, triglycerides and free fatty acids. There were significantly higher levels of total cholesterol and HDL-cholesterol in females compared to males and lower BMI and glucose levels in females (Table 1).

**Table 1:** **General characteristics of study population compared by sex and separated by ethnicity**

	**Caucasians**					**East Asians**				
	**Males**		**Females**		**p-value**	**Males**		**Females**		**p-value**
Population (#)	113	SEM	298	SEM		98	SEM	277	SEM	
Age (yrs)	23.1	0.2	23.1	0.1	0.87	22.4	0.2	22.1	0.1	0.26
BMI (kg/m^2^)	23.3	0.3	23.1	0.2	0.54	23.2	0.3	21.2	0.1	<0.01*
HOMA-IR	1.2	0.1	1.4	0.1	0.02*	1.4	0.1	1.5	0.1	0.84
Glucose (mmol/L)	4.9	0.1	4.7	<0.1	<0.01*	5.0	<0.1	4.7	<0.1	<0.01*
Insulin (pmol/L)	37.1	2.1	47.4	1.7	<0.01*	45.7	2.3	48.1	2.9	0.62
Total cholesterol (mmol/L)	4.0	0.1	4.4	0.1	<0.01*	4.1	0.1	4.3	<0.1	<0.05*
HDL-cholesterol (mmol/L)	1.4	<0.1	1.7	<0.1	<0.01*	1.4	<0.1	1.7	<0.1	<0.01*
LDL-cholesterol (mmol/L)	2.1	0.1	2.2	<0.1	0.32	2.3	0.1	2.2	<0.1	0.24
Triglycerides (mmol/L)	1.0	0.1	1.0	<0.1	0.86	1.0	0.1	1.0	<0.1	0.19
Free fatty acids (μmol/L)	454	24.6	489	14.2	0.21	453	21.4	521	15.4	0.02*
% Total energy from fat	27.4	0.6	27.9	0.4	0.56	26.3	0.5	26.2	0.3	0.98

Statistical differences were determined using Student’s t-test. The * denotes p-values which are significant.

*Abbreviation: SEM* standard error of the mean.

### PUFA concentrations and desaturation indices

#### N-6 PUFA

Concentrations of selected n-6 and n-3 PUFA are presented in Table 2. Caucasian and East Asian females had higher LA concentrations than males. Caucasian females had higher AA concentrations than Caucasian males. There were no significant differences in [AA/LA] desaturation indices between males and females in both ethnicities.

**Table 2:** **Estimates of fatty acid concentrations (μg/ml) in Caucasian and East Asian males and females**

**Fatty acid**	**Caucasians**						**East Asians**					
	**Males**		**Females**		**p-value**		**Males**		**Females**		**p-value**	
	**μg/ml**	**SD**	**μg/ml**	**SD**	**t-test**	**MLR**	**μg/ml**	**SD**	**μg/ml**	**SD**	**t-test**	**MLR**
18:2n6	594.5	151.9	641.6	141.0	<0.01*	<0.01*	626.6	159.6	669.2	139.1	0.01*	0.03*
18:3n3	14.1	9.2	15.2	7.0	0.18	0.20	14.4	6.8	15.7	6.5	0.10	0.03*
20:4n6	118.7	35.6	127.3	37.3	0.04*	0.09	112.7	31.5	114.1	30.8	0.71	0.88
20:5n3	11.5	7.5	11.7	8.2	0.82	<0.01*	12.9	7.7	12.8	9.9	0.95	0.80
22:6n3	23.7	9.0	31.3	12.5	<0.01*	<0.01*	32.3	11.2	35.2	12.1	0.03*	0.04*
[AA/LA]	0.204	0.005	0.201	0.003	0.60	0.24	0.197	0.012	0.176	0.004	0.09	0.19
[DHA/ALA]	2.0	1.1	2.3	1.1	0.02*	0.03*	2.5	1.2	2.5	1.1	0.85	0.47
[EPA/ALA]	0.923	0.062	0.851	0.038	0.32	0.30	1.003	0.070	0.906	0.048	0.29	0.14
[DHA/EPA]	2.5	1.1	3.3	1.5	<0.01*	<0.01*	3.0	1.0	3.7	1.7	<0.01*	<0.01*

Select fatty acids used as substrate and product of D5D and D6D desaturase indices. The * denotes p-values which are significant (<0.05). P-values were determined using Student’s t-test and multiple linear regression (MLR) models which were adjusted for BMI, age, dietary LA, ALA, EPA and DHA, % total energy from dietary fat and physical activity. Caucasian males: n = 113; Caucasian females: n = 298; East Asian Males: n = 98; East Asian Females: n = 277. *Abbreviation: SD* standard deviation.

#### N-3 PUFA

Females and males, in both ethnicities, had similar circulating plasma concentrations of ALA and EPA as well as similar [EPA/ALA] desaturase indices. Caucasian females had significantly higher [DHA/ALA] desaturase index compared to males. The [DHA/ALA] desaturase index was not significantly different between East Asian males and females. Females from both ethnicities had significantly higher concentrations of DHA and higher [DHA/EPA] desaturase indices compared to males (Table 2).

### SNP selection

Twenty SNP from the *FADS1/FADS2* gene cluster were selected. One SNP (rs740006) was identified as non-polymorphic in the 4 tested populations (Caucasian males, Caucasian females, East Asian males, and East Asian females). In Caucasian males 3 additional SNP were non-polymorphic: rs412334, rs695867, and rs2845573. In Caucasian females one additional SNP was non-polymorphic: rs695867. In East Asian males, 8 SNP (rs412334, rs695867, rs174611, rs174627, rs17831757, rs482548, rs498793 and rs968567) were non-polymorphic. In East Asian females, *FADS1* rs174547 and *FADS2* rs174570 and rs174576 were not in HWE. In addition, 7 SNP were non-polymorphic in East Asian females: rs412334, rs695867, rs174611, rs174627, rs17831757, rs482548, and rs968567. SNP identified as non-polymorphic or not in HWE were excluded from further analyses (Additional file 1: Table S1).

### Sex-specific effects of *FADS* SNP on desaturase indices up to AA and EPA

#### Caucasian males

Multiple linear regression analysis showed 2 associations between n-3 ADI and *FADS* genetic variants (rs174611 and rs526126) in Caucasian males; however, none withstood multiple testing.

Multiple linear regression analyses of the 19 SNP and n-6 ADI revealed significant associations with 13 SNP (rs174547, rs174570, rs174576, rs174602, rs174593, rs174611, rs174626, rs174627, rs17831757, rs2072114, rs2851682, rs526126, and rs968567) (Table 3); however, only 3 SNP (rs174547, rs174576, and rs174611) withstood multiple testing. Carriers of the minor alleles of all aforementioned SNP were observed to have lower ADI. Additional analyses including the SNP with the strongest association (rs174547, p = 1.57 × 10^−6^) as a covariate resulted in the loss of the association between n-6 ADI and rs174611. In addition, results suggested that effects of rs174547 and rs174576 were dependent on each other.

**Table 3:** **Estimates of aggregate desaturase indices (ADI) according to genotype for each SNP in Caucasian males**

	**n-3 ADI (EPA:ALA)**							**n-6 ADI (AA:LA)**						
	**MM**		**Mm**		**mm**		**p-value**	**MM**		**Mm**		**mm**		**p-value**
	**ADI**	**SEM**	**ADI**	**SEM**	**ADI**	**SEM**		**ADI**	**SEM**	**ADI**	**SEM**	**ADI**	**SEM**	
*FADS1*														
rs174547	1.130	0.110	0.841	0.083	0.569	0.083	0.23	0.234^a^	0.008	0.182^b^	0.007	0.146^b^	0.146	1.57 × 10^−6^*
*FADS2*														
rs174570	0.971	0.081	0.784	0.069	0.428		0.73	0.214^a^	0.006	0.180^b^	0.010	0.111^a,b^		0.02
rs174576	1.153	0.117	0.818	0.081	0.604	0.072	0.12	0.235^a^	0.008	0.194^b^	0.007	0.156^b^	0.015	0.10 × 10^−3^*
rs174579	1.026	0.089	0.766	0.073	0.629	0.165	0.14	0.216	0.006	0.188	0.010	0.149	0.029	0.07
rs174593	1.044	0.092	0.823	0.096	0.680	0.099	0.19	0.221^a^	0.007	0.188^b^	0.008	0.181^a,b^	0.023	0.02
rs174602	0.997	0.087	0.880	0.104	0.657	0.092	0.23	0.216	0.008	0.193	0.008	0.177	0.020	0.04
rs174611	1.088	0.112	0.802	0.063	0.679	0.114	0.02	0.228^a^	0.007	0.187^b^	0.008	0.164^b^	0.020	4.31 × 10^−6^*
rs174626	1.089	0.112	0.916	0.112	0.791	0.079	0.19	0.232^a^	0.010	0.203^a,b^	0.008	0.179^b^	0.009	3.20 × 10^−3^
rs174627	1.017	0.085	0.761	0.076	0.567	0.100	0.12	0.216^a^	0.006	0.186^b^	0.010	0.108^b^	0.022	3.90 × 10^−3^
rs17831757	0.957	0.077	0.829	0.077	0.842	0.605	0.40	0.211	0.006	0.185	0.010	0.149	0.059	0.03
rs2072114	0.971	0.077	0.872	0.119	0.487	0.024	0.80	0.216^a^	0.007	0.183^b^	0.009	0.164^a,b^	0.004	5.10 × 10^−3^
rs2851682	0.967	0.077	0.824	0.094	0.237		0.57	0.212	0.006	0.182	0.009	0.090		0.02
rs482548	0.903	0.062	1.096	0.227	0.494		0.71	0.202	0.006	0.219	0.013	0.192		0.39
rs498793	0.871	0.103	0.936	0.075	1.041	0.183	0.32	0.195	0.009	0.207	0.009	0.219	0.012	0.43
rs526126	1.042	0.089	0.765	0.074	0.514	0.053	0.01	0.219^a^	0.006	0.186^b^	0.010	0.145^b^	0.011	1.90 × 10^−3^
rs968567	1.026	0.083	0.720	0.078	0.467		0.15	0.216^a^	0.006	0.182^b^	0.010	0.130^a,b^		0.03

Results of analysis of 16 SNP from the *FADS* cluster for associations with n-3 and n-6 ADI. Different letters (^a^ or ^b^) denote values that are significantly different between groups. The * denotes p-values which are significant and withstood multiple testing. P-values were determined using multiple linear regression models which were adjusted for BMI, age, dietary LA, ALA, EPA and DHA, % total energy from dietary fat and physical activity. *Abbreviations: M* major, *m* minor, *ADI* aggregate desaturase indices, *SEM* standard error of the mean.

#### Caucasian females

Multiple linear regression analyses of the 19 SNP and n-3 ADI in Caucasian females revealed significant associations only between n-3 ADI and 7 SNP (rs174547, rs174576, rs174579, rs174626, rs174627, rs174593, and rs968567) (Table 4); however, adjustment for multiple testing resulted in the loss of all associations.

**Table 4:** **Estimates of aggregate desaturase indices (ADI) according to genotype for each SNP in Caucasian females**

	**n-3 ADI (EPA:ALA)**							**n-6 ADI (AA:LA)**						
	**MM**		**Mm**		**mm**		**p-value**	**MM**		**Mm**		**mm**		**p-value**
	**ADI**	**SEM**	**ADI**	**SEM**	**ADI**	**SEM**		**ADI**	**SEM**	**ADI**	**SEM**	**ADI**	**SEM**	
*FADS1*														
rs174547	0.987^a^	0.063	0.972^a,b^	0.053	0.533^b^	0.095	0.6 × 10^−3^	0.225^a^	0.004	0.189^b^	0.004	0.156^c^	0.006	1.00 × 10^−16^*
rs412334	0.861	0.048	0.821	0.069	0.732	0.098	0.43	0.197^b^	0.004	0.209^a,b^	0.006	0.243^a^	0.012	0.01
*FADS2*														
rs174570	0.886	0.044	0.769	0.082	0.428	0.046	0.09	0.212^a^	0.003	0.176^b^	0.006	0.133^b^	0.005	9.02 × 10^−11^*
rs174576	0.999^a^	0.064	0.791^b^	0.052	0.526^b^	0.092	0.4 × 10^−3^	0.226^a^	0.004	0.190^b^	0.004	0.156^c^	0.006	7.33 × 10^−17^*
rs174579	0.938^a^	0.054	0.686^b^	0.044	0.757^a,b^	0.196	4.2 × 10^−3^	0.211^a^	0.004	0.185^b^	0.005	0.184^a,b^	0.014	0.0001*
rs174593	0.910	0.054	0.797	0.061	0.677	0.125	0.04	0.213^a^	0.004	0.191^b^	0.004	0.166^b^	0.007	6.99 × 10^−6^*
rs174602	0.835	0.041	0.883	0.079	0.762	0.189	0.79	0.204^a^	0.004	0.202^a^	0.005	0.162^b^	0.008	7.40 × 10^−3^
rs174611	0.941	0.060	0.760	0.053	0.708	0.101	0.13	0.213^a^	0.004	0.189^b^	0.005	0.184^b^	0.010	9.32 × 10^−5^*
rs174626	1.001^a^	0.084	0.847^a,b^	0.053	0.691^b^	0.070	2.9 × 10^−3^	0.219^a^	0.007	0.202^a^	0.004	0.182^b^	0.006	1.47 × 10^−5^*
rs174627	0.895	0.046	0.747	0.076	0.474	0.061	8.7 × 10^−3^	0.207^a^	0.004	0.188^b^	0.006	0.157^b^	0.014	2.40 × 10^−3^
rs17831757	0.873	0.047	0.763	0.063	0.795	0.130	0.75	0.204	0.003	0.189	0.007	0.221	0.024	0.02
rs2072114	0.885	0.043	0.778	0.091	0.387	0.042	0.06	0.210^a^	0.003	0.180^b^	0.006	0.143^b^	0.008	1.23 × 10^−8^*
rs2845573	0.873	0.041	0.749	0.113	0.428	0.046	0.28	0.207^a^	0.003	0.172^b^	0.007	0.133^b^	0.005	1.58 × 10^−7^*
rs2851682	0.876	0.042	0.748	0.102	0.428	0.046	0.32	0.207^a^	0.003	0.177^b^	0.007	0.133^b^	0.005	1.65 × 10^−6^*
rs482548	0.840	0.044	0.879	0.081	0.989	0.356	0.64	0.198	0.003	0.215	0.007	0.197	0.022	0.05
rs498793	0.812	0.074	0.890	0.053	0.771	0.081	0.64	0.191	0.006	0.206	0.004	0.204	0.008	0.51
rs526126	0.873	0.049	0.823	0.070	0.653	0.086	0.57	0.205	0.004	0.192	0.005	0.195	0.014	0.09
rs968567	0.927^a^	0.051	0.687^b^	0.051	0.487^a,b^	0.079	0.02	0.209^a^	0.004	0.186^b^	0.004	0.168^a,b^	0.021	0.70 × 10^−3^

Results of analysis of 18 SNP from the *FADS* cluster for associations with n-3 and n-6 ADI. Different letters (^a^, ^b^ or ^c^) denote values that are significantly different between groups. The * denotes p-values which are significant and withstood multiple testing. P-values were determined using multiple linear regression models which were adjusted for BMI, age, dietary LA, ALA, EPA and DHA, % total energy from dietary fat and physical activity. *Abbreviations: M* major, *m* minor, *ADI* aggregate desaturase indices, *SEM* standard error of the mean.

Multiple linear regression analyses showed that 15 (rs174547, rs412334, rs174570, rs174576, rs174679, rs174593, rs174602, rs174611, rs174626, rs174627, rs17831757, rs2072114, rs2845573, rs2851682, and rs968567) of the 19 SNP analyzed were significantly associated with altered n-6 ADI (Table 4). Out of the 15 associations, 10 (rs174547, rs174570, rs174576, rs174679, rs174593, rs174611, rs174626, rs2072114, rs2845573, and rs2851682) withstood multiple testing. Carriers of the minor alleles of all aforementioned SNP displayed lower ADI.

Additional analyses including rs174576 (p = 7.33 × 10^−17^) as a covariate resulted in the loss of 5 (rs174593, rs174611, rs174626, rs2072114, rs2851682) of the previously identified associations (data not shown). In addition, results suggested that effects of rs174570, rs174679 and rs2845573 were independent.

#### East Asian males

No significant associations between SNP and n-3 or n-6 ADI were identified in East Asian males (Table 5).

**Table 5:** **Estimates of aggregate desaturase indices (ADI) according to genotype for each SNP in East Asian males**

	**n-3 ADI (EPA:ALA)**							**n-6 ADI (AA:LA)**						
	**MM**		**Mm**		**mm**		**p-value**	**MM**		**Mm**		**mm**		**p-value**
	**ADI**	**SEM**	**ADI**	**SEM**	**ADI**	**SEM**		**ADI**	**SEM**	**ADI**	**SEM**	**ADI**	**SEM**	
*FADS1*														
rs174547	1.036	0.168	0.963	0.078	1.173	0.158	0.41	0.198	0.050	0.182	0.005	0.240	0.013	0.28
*FADS2*														
rs174570	1.036	0.168	0.963	0.078	1.173	0.158	0.41	0.198	0.050	0.182	0.005	0.240	0.013	0.28
rs174576	1.026	0.162	0.982	0.079	1.152	0.165	0.51	0.198	0.050	0.184	0.005	0.239	0.013	0.41
rs174579	0.972	0.078	1.156	0.172	1.534	0.781	0.58	0.207	0.022	0.182	0.008	0.143	0.033	0.88
rs174593	0.966	0.077	1.182	0.177	1.534	0.781	0.56	0.205	0.022	0.184	0.008	0.143	0.033	0.90
rs174602	0.991	0.102	1.130	0.120	0.872	0.172	0.63	0.209	0.010	0.173	0.005	0.240	0.082	0.13
rs174626	1.078	0.111	1.011	0.108	0.890	0.213	0.23	0.194	0.010	0.209	0.033	0.166	0.012	0.78
rs2072114	1.144	0.109	1.028	0.114	0.830	0.170	0.41	0.217	0.010	0.176	0.005	0.223	0.083	0.34
rs2845573	1.123	0.104	1.051	0.121	0.816	0.161	0.31	0.214	0.010	0.175	0.005	0.222	0.078	0.41
rs2851682	1.144	0.109	1.038	0.116	0.817	0.161	0.42	0.217	0.010	0.177	0.005	0.218	0.078	0.42
rs526126	1.044	0.081	1.073	0.185	0.652	0.218	0.53	0.194	0.006	0.229	0.070	0.141	0.017	0.86

Results of analysis of 11 SNP from the *FADS* cluster for associations with n-3 and n-6 ADI. P-values were determined using multiple linear regression models which were adjusted for BMI, age, dietary LA, ALA, EPA and DHA, % total energy from dietary fat and physical activity. *Abbreviations: M* major, *m* minor, *ADI* aggregate desaturase indices, *SEM* standard error of the mean.

#### East Asian females

Significant associations between SNP (rs2072114, rs2851682, and rs2845573) and n-3 ADI were identified in East Asian females; however, none withstood multiple testing. 7 SNP (rs174679, rs174593, rs174602, rs174626, rs2072114, rs2845573 and rs2851682) were strongly associated with altered n-6 ADI (Table 6) and 5 SNP (rs174602, rs174626, rs2072114, rs2845573, and rs2851682) withstood multiple testing. Carriers of the minor alleles of all aforementioned SNP displayed lower ADI.

**Table 6:** **Estimates of aggregate desaturase indices (ADI) according to genotype for each SNP in East Asian females**

	**n-3 ADI (EPA:ALA)**							**n-6 ADI (AA:LA)**						
	**MM**		**Mm**		**mm**		**p-value**	**MM**		**Mm**		**mm**		**p-value**
	**ADI**	**SEM**	**ADI**	**SEM**	**ADI**	**SEM**		**ADI**	**SEM**	**ADI**	**SEM**	**ADI**	**SEM**	
*FADS2*														
rs174579	0.932	0.052	0.869	0.123	0.519	0.178	0.53	0.183	0.006	0.158	0.005	0.130	0.009	0.03
rs174593	0.940	0.052	0.849	0.125	0.587	0.154	0.57	0.183^a^	0.006	0.156^b^	0.005	0.131^a,b^	0.008	0.01
rs174602	0.995	0.072	0.867	0.081	0.797	0.122	0.27	0.199^a^	0.009	0.163^b^	0.003	0.148^b^	0.006	4.11 × 10^−5^*
rs174626	0.996	0.070	0.798	0.053	0.986	0.254	0.20	0.194^a^	0.009	0.162^b^	0.004	0.154^b^	0.007	0.30 × 10^−3^*
rs2072114	1.068^a^	0.100	0.889^a,b^	0.064	0.695^b^	0.086	0.01	0.209^a^	0.011	0.165^b^	0.003	0.142^b^	0.004	7.80 × 10^−8^*
rs2845573	1.065^a^	0.100	0.887^a,b^	0.063	0.702^b^	0.087	0.03	0.209^a^	0.011	0.165^b^	0.003	0.142^b^	0.004	3.82 × 10^−7^*
rs2851682	1.061^a^	0.100	0.886^a,b^	0.064	0.712^b^	0.087	0.04	0.208^a^	0.011	0.165^b^	0.0003	0.144^b^	0.005	1.24 × 10^−6^*
rs498793	0.925	0.055	0.802	0.108	1.177	0.614	0.55	0.177	0.006	0.165	0.006	0.203	0.016	0.35
rs526126	0.935	0.065	0.862	0.074	0.844	0.198	0.68	0.183	0.007	0.163	0.004	0.151	0.010	0.16

Results of analysis of 9 SNP from the *FADS* cluster for associations with n-3 and n-6 ADI. Different letters (^a^ or ^b^) denote values that are significantly different between groups. The * denotes p-values which are significant and withstood multiple testing. P-values were determined using multiple linear regression models which were adjusted for BMI, age, dietary LA, ALA, EPA and DHA, % total energy from dietary fat and physical activity. *Abbreviations: M* major, *m* minor, *ADI* aggregate desaturase indices, *SEM* standard error of the mean.

Results from multiple linear regression analyses including rs2072114 (p = 7.80 × 10^−8^) as a covariate suggested that effects of rs174602, rs2845573, or rs2851682 are dependent on rs2072114 (data not shown).

### DHA synthesis from EPA and influence of *FADS2* SNP

Associations between *FADS* polymorphisms and [DHA/ALA] desaturase index were determined but no significant associations withstood multiple testing (data not shown).

#### Caucasian population

Multiple linear regression analyses of [DHA/EPA] desaturase index and *FADS2* SNP were performed. Only rs526126 was significantly associated with altered [DHA/EPA] in Caucasian males; however, the association did not withstand multiple testing. In Caucasian females rs174570, rs174576, rs174593, rs2072114 and rs2845573 were significantly associated with altered [DHA/EPA] desaturase index (Table 7). rs174576 displayed the strongest association with the [DHA/EPA] desaturase index (p < 0.0001) and was the only SNP that remained significant after adjustment for multiple testing. The minor allele, A, was associated with increased [DHA/EPA] desaturase index in Caucasian females.

**Table 7:** **Estimates of D6D activity according to genotype for each** ***FADS2*** **SNP in Caucasians**

	**Males**							**Females**						
	**MM**		**Mm**		**mm**		**p-value**	**MM**		**Mm**		**mm**		**p-value**
		**SEM**		**SEM**		**SEM**			**SEM**		**SEM**		**SEM**	
rs174570	2.461	0.118	2.461	0.118	3.865		0.60	3.121^b^	0.100	3.546^a,b^	0.182	5.180^a^	0.612	0.60 × 10^−2^
rs174576	2.340	0.157	2.543	0.168	2.980	1.138	0.52	2.984^b^	0.108	3.254^b^	0.133	4.388^a^	0.334	4.44 × 10^−5^*
rs174579	2.437	0.134	2.625	0.203	2.625	0.203	0.43	3.108	0.101	3.551	0.177	3.447	0.445	0.12
rs174593	2.524	0.159	2.516	0.169	2.349	0.276	0.68	3.074	0.101	3.529	0.168	3.343	0.341	0.03
rs174602	2.519	0.154	2.453	0.170	2.698	0.346	0.71	3.149	0.099	3.384	0.168	3.789	0.537	0.15
rs174611	2.52	0.196	2.329	0.153	3.160	0.353	0.07	3.152	0.123	3.484	0.148	2.995	0.231	0.59
rs174626	2.417	0.198	2.512	0.167	2.605	0.215	0.72	3.118	0.169	3.200	0.119	3.564	0.203	0.30
rs174627	2.443	0.129	2.638	0.215	2.859	0.123	0.50	3.245	0.102	3.346	0.192	3.212	0.517	0.90
rs17831757	2.531	0.125	2.352	0.221	3.131	1.429	0.73	3.282	0.105	3.212	0.177	3.332	0.285	0.70
rs2072114	2.460	0.128	2.587	0.215	3.000	1.030	0.91	3.144^b^	0.095	3.444^b^	0.212	5.370^a^	0.460	1.20 × 10^−3^
rs2845573	NA							3.169^b^	0.094	3.585^a,b^	0.246	5.179^a^	0.612	0.03
rs2851682	2.435	0.113	2.708	0.297	4.560		0.15	3.208	0.098	3.354	0.208	5.180	0.612	0.07
rs482548	2.537	0.116	2.345	0.324	2.958		0.90	3.303	0.102	3.142	0.171	2.233	0.206	0.38
rs498793	2.759	0.195	2.305	0.127	2.388	0.259	0.38	3.445	0.168	3.051	0.102	3.665	0.296	0.06
rs526126	2.398	0.117	2.589	0.234	3.528	0.581	0.02	3.250	0.107	3.377	0.182	2.967	0.274	0.43
rs968567	2.457	0.128	2.619	0.214	2.982		0.61	3.162	0.100	3.506	0.185	3.463	0.703	0.32

Results of analysis of 16 SNP from the *FADS2* for associations with D6D activity index [DHA/EPA]. Different letters (^a^ or ^b^) denote values that are significantly different between groups. The * denotes p-values which are significant and withstood multiple testing. P-values were determined using multiple linear regression models which were adjusted for BMI, age, dietary LA, ALA, EPA and DHA, % total energy from dietary fat and physical activity. *Abbreviations: D6D* delat-6-desaturase, *M* major, *m* minor, *SEM* standard error of the mean, *NA* not applicable.

#### Asian population

In East Asian males, 3 SNP (rs2072114, rs2851682 and rs526126) were significantly associated with altered [DHA/EPA] desaturase index (Table 8); however, none of the associations withstood multiple testing.

**Table 8:** **Estimates of D6D activity according to genotype for each** ***FADS2*** **SNP in East Asians**

	**Males**							**Females**						
	**MM**		**Mm**		**mm**		**p-value**	**MM**		**Mm**		**mm**		**p-value**
		**SEM**		**SEM**		**SEM**			**SEM**		**SEM**		**SEM**	
rs174570	3.234	0.270	3.042	0.179	2.573	0.197	0.14	NA						
rs174576	3.288	0.266	2.975	0.175	2.609	0.204	0.11	NA						
rs174579	3.053	0.160	2.987	0.208	1.722	0.129	0.52	3.516	0.127	3.978	0.200	3.735	0.866	0.32
rs174593	3.059	0.158	2.966	0.217	1.722	0.129	0.52	3.507	0.125	4.027	0.208	3.488	0.715	0.23
rs174602	2.938	0.174	2.819	0.205	3.572	0.322	0.06	3.490	0.176	3.568	0.143	4.185	0.279	0.13
rs174626	3.034	0.214	3.056	0.172	2.607	0.436	0.93	3.334	0.140	3.906	0.176	3.829	0.324	0.05
rs2072114	2.628^b^	0.137	3.074^a,b^	0.198	3.526^a^	0.373	0.03	3.221^b^	0.130	3.630^a,b^	0.185	4.322^a^	0.233	0.50 × 10^−3^
rs2845573	2.693	0.145	3.024	0.207	3.537	0.353	0.06	3.231^b^	0.129	3.644^a,b^	0.183	4.329^a^	0.237	1.20 × 10^−3^
rs2851682	2.628^b^	0.137	3.066^a,b^	0.203	3.522^a^	0.353	0.04	3.231^b^	0.130	3.659^a,b^	0.185	4.291^a^	0.230	1.80 × 10^−3^
rs498793	NA							3.606	0.118	3.885	0.262	2.837	0.690	0.32
rs526126	2.819	0.132	3.382	0.340	3.968	0.570	0.05	3.601	0.124	3.758	0.220	3.296	0.403	0.94

Results of analysis of 11 SNP from *FADS2* for associations with D6D activity index [DHA/EPA]. Data represented as Mean ± SEM. Different letters (^a^ or ^b^) denote values that are significantly different between groups. P-values were determined using multiple linear regression models which were adjusted for BMI, age, dietary LA, ALA, EPA and DHA, % total energy from dietary fat and physical activity. *Abbreviations: D6D* delat-6-desaturase, *M* major, *m* minor, *SEM* standard error of the mean, *NA* not applicable.

In East Asian females, 3 SNP (rs2072114, rs2845573 and rs2851682) were significantly associated with [DHA/EPA] desaturase index (Table 8) but none withstood multiple testing.

### Influence of hormonal contraceptives on PUFA concentrations and desaturation indices

**Figure 1:**
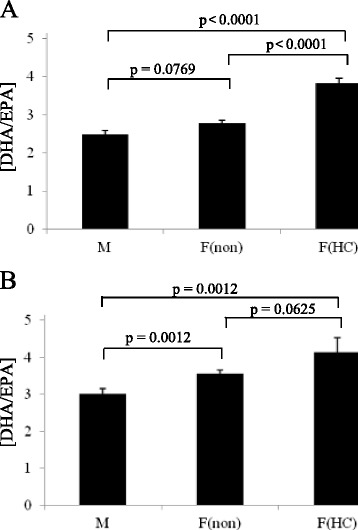
[DHA/EPA] desaturase indices in males (M) compared to females who are not using HC, F(non), and females who are using HC, F(HC). Differences where determined in Caucasians **(A)** and East Asians **(B)**. Data presented as mean ± standard error mean. P-values were determined using multiple linear regression models which were adjusted for BMI, age, dietary LA, ALA, EPA and DHA, % total energy from dietary fat and physical activity. Caucasian M: n = 113; Caucasian F(non): n = 161; Caucasian F(HC): n = 137; East Asian M: n = 98; East Asian F(non): n = 234; East Asian F(HC): n = 43.

Caucasian females using HC had significantly higher [AA/LA], [EPA/ALA] and [DHA/EPA] desaturase indices, and DHA concentrations. There was no significant difference in [DHA/ALA] desaturase index in female users and non-users of HC (Table 9). Among East Asian females, there were no significant differences in [AA/LA] desaturase index, [DHA/ALA] desaturase index, [EPA/ALA] desaturase index or DHA concentrations. Users of HC tended to have higher [DHA/EPA] desaturase index than non-users; however, the effect of HC use was not significant (Table 9, Figure 1).

**Table 9:** **Estimates of fatty acid concentrations (μg/ml) in Caucasian and Asian females using hormonal contraceptives**

**Fatty acid**	**Caucasian females**					**East Asian females**				
	**non (n = 161)**		**HC (n = 137)**		**p-value**	**non (n = 234)**		**HC (n = 43)**		**p-value**
	**μg/ml**	**SD**	**μg/ml**	**SD**		**μg/ml**	**SD**	**μg/ml**	**SD**	
18:2n6	616.5	138.1	671.1	139.0	<0.01	664.0	140.6	698.0	128.1	<0.05*
18:3n3	14.0	7.3	16.6	6.3	<0.01*	15.3	6.1	17.8	7.7	0.05
20:4n6	115.9	34.1	140.7	36.6	<0.01*	112.4	30.4	123.1	31.6	<0.01*
20:5n3	12.0	8.7	11.3	7.5	0.42	12.6	9.6	14.0	11.3	0.45
22:6n3	27.7	12.1	35.4	11.7	<0.01*	34.8	11.5	37.5	14.7	0.23
[AA/LA]	0.190	0.004	0.213	0.005	<0.01*	0.176	0.005	0.179	0.007	0.81
[DHA/ALA]	2.3	1.1	2.4	1.0	0.42	2.5	1.1	2.4	1.1	0.38
[EPA/ALA]	0.944	0.055	0.741	0.049	<0.01*	0.910	0.052	0.890	0.119	0.89
[DHA/EPA]	2.8	1.2	3.8	1.6	<0.01*	3.6	1.5	4.1	2.6	0.06

Select fatty acids used as substrate and product of D5D and D6D desaturase indices. The * denotes p-values which are significant. P-values were determined using multiple linear regression models which were adjusted for BMI, age, dietary LA, ALA, EPA and DHA, % total energy from dietary fat and physical activity. Caucasian females: n = 298; East Asian Females: n = 277. *Abbreviation: SD* standard deviation.

### Relationship between HC and SNP

**Figure 2:**
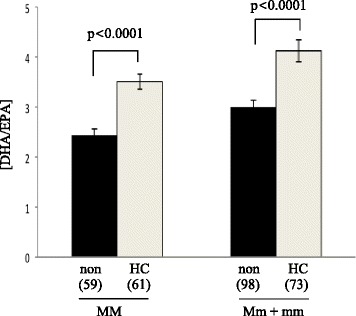
Effects of HC use on [DHA/EPA] desaturase indices in Caucasian females classified by genotype of rs174576 SNP. Results demonstrated that the significant effect of HC use is independent of genotype. Data presented as mean ± standard error mean. P-values were determined using multiple linear regression models which were adjusted for BMI, age, dietary LA, ALA, EPA and DHA, % total energy from dietary fat and physical activity. MM: major homozygous; Mm + mm: heterozygous + minor homozygous.

To explore the possibility of an interaction between HC and *FADS2* SNP, multiple linear regression models (that were adjusted for age, BMI, dietary intake of LA, ALA, EPA and DHA, % of total energy from dietary fat and physical activity) were built to include the SNP rs174576, which was significantly associated with altered [DHA/EPA] desaturase indices, as a covariate. This enabled the determination of the effect of hormonal contraceptive use as well any interaction between genotype and HC use. In Caucasian females, the association between [DHA/EPA] desaturase indices and rs174576 or hormonal contraceptive use remained significant (p = 0.0009 and p < 0.0001, respectively), suggesting that rs175476 and HC use independently influence [DHA/EPA] desaturase indices (data not shown). In addition there was no interaction between effects of genotype and HC use on [DHA/EPA] desaturase indices (rs174576*HC, p = 0.74, data not shown). Furthermore, when Caucasian females were stratified by genotype (homozygous for major allele of rs174576 *vs.* heterozygous and minor homozygous) [DHA/EPA] desaturase indices remained significantly higher in females using HC confirming that the effect of HC use on [DHA/EPA] desaturase indices is independent of genotype (Figure 2).

## Discussion

In this study, we undertook a comprehensive examination of major factors that influence the metabolism of essential fatty acids from precursor to product. Sex, ethnicity and *FADS* polymorphisms have been shown to play important roles influencing the conversion of ALA to EPA and LA to AA. However, the present study revealed that genetic polymorphisms have less influence in the metabolism of EPA to DHA while HC use plays a prominent role in that process, specifically in Caucasian women. These findings highlight the differential roles of sex, ethnicity, and *FADS* polymorphisms within discrete steps in the metabolic conversion of essential fatty acids.

### Role of *FADS* polymorphisms on [EPA/ALA] and [AA/LA] desaturation indices

Previously, we showed that *FADS1* and *FADS2* genetic variants alter desaturase indices in Caucasians and East Asian Canadian adults [20]. These analyses were examined in a smaller mixed gender subset of the TNH subjects (147 subjects) while the present study examined sex-specific associations in 786 subjects that met our inclusion and exclusion criteria. Findings from the present study confirmed significant associations previously reported by our group showing that n-6 ADI are associated with *FADS1* and *FADS2* polymorphisms (rs174547, rs174576) in Caucasians. Using the SNP Annotation and Proxy Search (SNAP) database, rs174547 and rs174576 were found to be in high linkage disequilibrium [20]. We also confirmed that rs2072114 is strongly associated with n-6 ADI in East Asians; however, examination of sex-dependent associations in this study, revealed that the rs2072114-ADI association is only significant in females. An additional observation is that the minor alleles of the identified *FADS* SNP (rs174547, rs174570, rs174576, rs174679, rs174593, rs174602, rs174611, rs174626, rs2072114, rs2845573, rs2851682) were associated with lower [AA/LA], but not [EPA/ALA], desaturation indices. The potential significance for carriers of the minor alleles are lower [AA/LA] and higher [EPA/ALA] indices, subsequently, lower risk of inflammatory diseases associated with the production of lower amounts of AA and higher amounts of EPA derived inflammatory and anti-inflammatory eicosanoids, respectively [17]. *FADS* polymorphisms have been shown to explain 28% of variability in AA levels in serum phospholipid fatty acids of Caucasian participants [25]. AA is a primary precursor for the synthesis of pro-inflammatory eicosanoids as well as the vasoconstrictor TXB_2_, and AA-generated prostaglandins are shown to be pro-arrhythmic [26-28]. Taken together, the identified association between SNP and altered n-6 ADI in females may contribute to modified risk of inflammatory and cardiovascular conditions through the alteration of circulating concentrations of AA. We acknowledge that a limitation of our study population is that it is comprised of young healthy Canadian adults but differences of circulating fatty acids may be important early biomarkers in chronic disease prevention.

### Role of *FADS2* polymorphisms in altered [DHA/EPA] desaturation index and DHA concentrations between sexes and ethnicities

Previously, the impact of *FADS* polymorphisms on D5D and D6D desaturase indices was investigated in regard to AA and EPA synthesis [20]. However, D6D is also involved in the synthesis of DHA from EPA [5]. Therefore, we sought to determine the role of *FADS2* genetic variations on D6D desaturase index in regard to subsequent conversion of EPA to DHA. Others have reported significant associations between *FADS1/FADS2* polymorphisms with n-6 and n-3 fatty acids except for DHA [15,25]. The present study identifies a significant association of one *FADS2* SNP with the [DHA/EPA] desaturase index specific to Caucasian females. Carriers of the minor allele of rs174576 had higher [DHA/EPA] desaturase indices. It is important to note that there was no difference in [EPA/ALA] desaturase indices in females compared to males. Thus, higher DHA concentrations in Caucasian and East Asian females are mainly attributed to the conversion of EPA to DHA. It is worth noting that SNP of elongases likely have an effect on concentrations of polyunsaturated fatty acids [29]; however, further research is needed to determine sex-, ethnic-, and hormonal-specific effects of genetic variations of elongases.

### Role of hormonal contraceptives use in altered desaturase indices and DHA concentrations in females

In addition to the role of *FADS* polymorphisms, results from the present study demonstrated the positive effect of HC use on DHA concentrations and [DHA/EPA] desaturase indices in females. HC use had no effect on [DHA/ALA] desaturase index but had an effect on [EPA/ALA]. A role for HC in modifying DHA concentration is possible. Sex hormones, presumably estrogen via increasing peroxisome proliferator-activated receptor -alpha activity (reviewed in [30]), were identified as the driving factor in the more efficient conversion of ALA to DHA in females [12]. Previously, increased DHA synthesis was observed in females using HC and, more recently, HC use was positively correlated with percent DHA of total fatty acids in red blood cell of Icelandic women [18,19]. In this regard, our study showed significantly higher [DHA/EPA] desaturase indices in females, and in particular, HC users, relative to males (Figure 2). East Asian HC users and non users were not different, however, a larger sample size is needed to verify this finding as there were only a limited number of (n = 43) East Asian HC users in this study. Results showed that the absolute concentration of DHA is differentially influenced between ethnicities (East Asians and Caucasians). There was no significant difference in absolute DHA concentrations between HC users and non users in East Asian females. Furthermore, *FADS* polymorphisms did not affect [DHA/EPA] desaturase indices in East Asian females. However, both HC and *FADS* polymorphisms were significantly associated with altered [DHA/EPA] desaturase indices in Caucasian females. Further analyses revealed the independent roles of the two covariates (hormonal contraceptives and SNP) in altering [DHA/EPA] desaturase indices in Caucasians. A probable interpretation is that the positive effect of HC on DHA synthesis was negated by a maximum threshold achieved through diet. This possibility was suggested by Pawlosky et al. reporting that a fish-based diet inhibited DHA synthesis, thus revealing the possibility of a potential feedback system for *de novo* synthesis of DHA [31]. It can also be argued that the lower concentrations of DHA in Caucasians, as a result of lower dietary DHA intake, allowed for the significant effect of both *FADS2* SNP and hormonal contraceptives to be unmasked. It is noteworthy that further stratification of HC users by type of HC used was not performed. A study examining effects of HC use on plasma proteomic biomarkers in TNH study subjects found that only the use of HC significantly altered the proteomic profile while the type of HC or duration of use had no significant effect [32]. The study also found that some of the proteins affected by HC use were biomarkers of dysregulation of inflammation, which is consistent with other reports [32-34]. DHA has been shown to be protective against a range of chronic diseases; therefore, it is important to understand factors that may render some women more susceptible to low levels of this fatty acid. Furthermore, DHA has a known role in cognitive development; thus, it is necessary to identify factors (such as ethnicity, *FADS* polymorphisms, or history of HC use) that may influence mothers circulating levels of DHA that is passed to their offspring.

### Strengths, weaknesses, and overall conclusions

We acknowledge the following limitations in this study: First, the use of plasma samples for measurement of endogenous fatty acids concentrations. Red blood cells and adipose tissue reflect longer term dietary fatty acid intake; however, fasting plasma fatty acids as an accessible tissue correlate with adipose tissue fatty acids [35]. Plasma fatty acids levels are determined by hepatic synthesis and diet; however, since subjects fasted overnight prior to sample collection, contribution of diet to plasma fatty acids concentrations is expected to be insignificant. Therefore, confounding by recent dietary fat intake is minimized. We also acknowledge that phospholipids are more frequently used for the investigation of polyunsaturated fatty acids (PUFA); however, studies that measure fatty acids from phospholipids often report and use percentage composition. In this study we have determined fatty acids concentrations (as opposed to percentage composition values), therefore, provided a quantitative perspective of specific circulating fatty acids, rather than relative changes that are dependent on levels of other fatty acids. The use of the desaturase index as an indirect measure of fatty acid conversion has been widely utilized [13,36-38]. While endogenous conversion by desaturases is recognized to be low, the desaturase index appears to be sufficiently sensitive across a number of studies [13,36-38].

## Conclusions

This study identified sex- and ethnic-specific effects of *FADS* polymorphisms on desaturase indices (Table 10); thus, demonstrating the importance of stratification of sample populations investigated by sex, ethnicity, genetics and contraceptive use. The increasing recognition that n-6 and n-3 PUFA influence chronic disease risk validate the need for better understanding of factors influencing circulating levels of these fatty acids.

**Table 10:** **A summary of the relationship between minor allelic SNP and HC on the conversion of LA to AA, ALA to EPA and EPA to DHA**

**Conversion**	**Effect (SNPs or HC)**	**Caucasians**		**Asians**	
		**Males**	**Females**	**Males**	**Females**
LA to AA	FADS1/FADS2 SNP	(−)	(−)	n/s	(−)
	HC	*n/a*	(+)	*n/a*	n/s
ALA to EPA	FADS1/FADS2 SNP	n/s	n/s	n/s	n/s
	HC	*n/a*	(+)	*n/a*	n/s
EPA to DHA	FADS2 SNP	n/s	(+)	n/s	n/s
	HC	*n/a*	(+)	*n/a*	n/s

Given that the findings of the present study can only show a relationship exists and not causality, (+) and (−) signs are used to denote relative differences in conversion (high and low, respectively) indirectly measured as a ratio of product to precursor fatty acid. *Abbreviations: HC* hormonal contraceptives, *SNP* single nucleotide polymorphisms, *n/a* not applicable, *n/s* no significant effect.

## Additional file

Additional file 1: Table S1. HWE values of *FADS1* and *FADS2* and their genotype frequencies.

## Abbreviations

*FADS1/2*: Fatty acid desaturase1/2
D5D: Delta-5-desaturase
D6D: Delta-6-desaturase
PUFA: Polyunsaturated fatty acids
LA: Linoleic acid
AA: Arachidonic acid
ALA: α-linolenic acid
EPA: Eicosapentaenoic acid
DHA: Docosahexaenoic acid
SNP: Single nucleotide polymorphisms
HC: Hormonal contraceptives
TNH: Toronto Nutrigenomics and Health
ADI: Aggregate desaturase indices
SEM: Standard error mean
M: Major allele
m: Minor allele

## References

[1] Cho HP, Nakamura MT, Clarke SD (1999). Cloning, expression, and nutritional regulation of the mammalian Delta-6 desaturase. J Biol Chem.

[2] Cho HP, Nakamura M, Clarke SD (1999). Cloning, expression, and fatty acid regulation of the human delta-5 desaturase. J Biol Chem.

[3] Nakamura MT, Nara TY (2004). Structure, function, and dietary regulation of delta6, delta5, and delta9 desaturases. Annu Rev Nutr.

[4] Marquardt A, Stohr H, White K, Weber BH (2000). cDNA cloning, genomic structure, and chromosomal localization of three members of the human fatty acid desaturase family. Genomics.

[5] Sprecher H (2000). Metabolism of highly unsaturated n-3 and n-6 fatty acids. Biochim Biophys Acta.

[6] Harris W (2010). Omega-6 and omega-3 fatty acids: partners in prevention. Curr Opin Clin Nutr Metab Care.

[7] Burdge GC, Calder PC (2006). Dietary alpha-linolenic acid and health-related outcomes: a metabolic perspective. Nutr Res Rev.

[8] Ruxton CH, Calder PC, Reed SC, Simpson MJ (2005). The impact of long-chain n-3 polyunsaturated fatty acids on human health. Nutr Res Rev.

[9] Spector AA (2009). Arachidonic acid cytochrome P450 epoxygenase pathway. J Lipid Res.

[10] Serhan CN (2005). Lipoxins and aspirin-triggered 15-epi-lipoxins are the first lipid mediators of endogenous anti-inflammation and resolution. Prostaglandins Leukot Essent Fatty Acids.

[11] Merino DM, Ma DW, Mutch DM (2010). Genetic variation in lipid desaturases and its impact on the development of human disease. Lipids Health Dis.

[12] Giltay EJ, Gooren LJ, Toorians AW, Katan MB, Zock PL (2004). Docosahexaenoic acid concentrations are higher in women than in men because of estrogenic effects. Am J Clin Nutr.

[13] Bokor S, Dumont J, Spinneker A, Gonzalez-Gross M, Nova E, Widhalm K (2010). Single nucleotide polymorphisms in the FADS gene cluster are associated with delta-5 and delta-6 desaturase activities estimated by serum fatty acid ratios. J Lipid Res.

[14] Koletzko B, Lattka E, Zeilinger S, Illig T, Steer C (2011). Genetic variants of the fatty acid desaturase gene cluster predict amounts of red blood cell docosahexaenoic and other polyunsaturated fatty acids in pregnant women: findings from the Avon Longitudinal Study of Parents and Children. Am J Clin Nutr.

[15] Gillingham LG, Harding SV, Rideout TC, Yurkova N, Cunnane SC, Eck PK (2013). Dietary oils and FADS1-FADS2 genetic variants modulate [13C]alpha-linolenic acid metabolism and plasma fatty acid composition. Am J Clin Nutr.

[16] Kim OY, Lim HH, Yang LI, Chae JS, Lee JH (2011). Fatty acid desaturase (FADS) gene polymorphisms and insulin resistance in association with serum phospholipid polyunsaturated fatty acid composition in healthy Korean men: cross-sectional study. Nutr Metab (Lond).

[17] Roke K, Ralston JC, Abdelmagid S, Nielsen DE, Badawi A, El-Sohemy A (2013). Variation in the FADS1/2 gene cluster alters plasma n-6 PUFA and is weakly associated with hsCRP levels in healthy young adults. Prostaglandins Leukot Essent Fatty Acids.

[18] Magnusardottir AR, Steingrimsdottir L, Thorgeirsdottir H, Gunnlaugsson G, Skuladottir GV (2009). Docosahexaenoic acid in red blood cells of women of reproductive age is positively associated with oral contraceptive use and physical activity. Prostaglandins Leukot Essent Fatty Acids.

[19] Burdge GC, Wootton SA (2002). Conversion of alpha-linolenic acid to eicosapentaenoic, docosapentaenoic and docosahexaenoic acids in young women. Br J Nutr.

[20] Merino DM, Johnston H, Clarke S, Roke K, Nielsen D, Badawi A (2011). Polymorphisms in FADS1 and FADS2 alter desaturase activity in young Caucasian and Asian adults. Mol Genet Metab.

[21] Fontaine-Bisson B, Wolever TM, Connelly PW, Corey PN, El-Sohemy A (2009). NF-kappaB -94Ins/Del ATTG polymorphism modifies the association between dietary polyunsaturated fatty acids and HDL-cholesterol in two distinct populations. Atherosclerosis.

[22] Matthews DR, Connolly AA, Holman RR, Turner RC (1985). Physiology of insulin secretion: problems of quantity and timing. Neth J Med.

[23] Lee IM, Paffenbarger RS (1998). Physical activity and stroke incidence: the Harvard Alumni Health Study. Stroke.

[24] Folch J, LEES M, SLOANE STANLEY GH (1957). A simple method for the isolation and purification of total lipides from animal tissues. J Biol Chem.

[25] Schaeffer L, Gohlke H, Muller M, Heid IM, Palmer LJ, Kompauer I (2006). Common genetic variants of the FADS1 FADS2 gene cluster and their reconstructed haplotypes are associated with the fatty acid composition in phospholipids. Hum Mol Genet.

[26] Bagga D, Wang L, Farias-Eisner R, Glaspy JA, Reddy ST (2003). Differential effects of prostaglandin derived from omega-6 and omega-3 polyunsaturated fatty acids on COX-2 expression and IL-6 secretion. Proc Natl Acad Sci U S A.

[27] Weber PC, Fischer S, von SC, Lorenz R, Strasser T (1986). The conversion of dietary eicosapentaenoic acid to prostanoids and leukotrienes in man. Prog Lipid Res.

[28] Li Y, Kang JX, Leaf A (1997). Differential effects of various eicosanoids on the production or prevention of arrhythmias in cultured neonatal rat cardiac myocytes. Prostaglandins.

[29] Tanaka T, Shen J, Abecasis GR, Kisialiou A, Ordovas JM, Guralnik JM (2009). Genome-wide association study of plasma polyunsaturated fatty acids in the InCHIANTI Study. PLoS Genet.

[30] Kitson AP, Stroud CK, Stark KD (2010). Elevated production of docosahexaenoic acid in females: potential molecular mechanisms. Lipids.

[31] Pawlosky RJ, Hibbeln JR, Lin Y, Goodson S, Riggs P, Sebring N (2003). Effects of beef- and fish-based diets on the kinetics of n-3 fatty acid metabolism in human subjects. Am J Clin Nutr.

[32] Josse AR, Garcia-Bailo B, Fischer K, El-Sohemy A (2012). Novel effects of hormonal contraceptive use on the plasma proteome. PLoS One.

[33] Piltonen T, Puurunen J, Hedberg P, Ruokonen A, Mutt SJ, Herzig KH (2012). Oral, transdermal and vaginal combined contraceptives induce an increase in markers of chronic inflammation and impair insulin sensitivity in young healthy normal-weight women: a randomized study. Hum Reprod.

[34] Divani AA, Luo X, Brandy KR, Meyer RM, Joseph MS, Flaherty JD (2012). Oral versus vaginal combined hormonal contraceptives’ effect on coagulation and inflammatory biomarkers among young adult women. Clin Appl Thromb Hemost.

[35] Baylin A, Kim MK, Donovan-Palmer A, Siles X, Dougherty L, Tocco P (2005). Fasting whole blood as a biomarker of essential fatty acid intake in epidemiologic studies: comparison with adipose tissue and plasma. Am J Epidemiol.

[36] Warensjo E, Ohrvall M, Vessby B (2006). Fatty acid composition and estimated desaturase activities are associated with obesity and lifestyle variables in men and women. Nutr Metab Cardiovasc Dis.

[37] Kroger J, Zietemann V, Enzenbach C, Weikert C, Jansen EH, Doring F (2011). Erythrocyte membrane phospholipid fatty acids, desaturase activity, and dietary fatty acids in relation to risk of type 2 diabetes in the European Prospective Investigation into Cancer and Nutrition (EPIC)-Potsdam Study. Am J Clin Nutr.

[38] Zietemann V, Kroger J, Enzenbach C, Jansen E, Fritsche A, Weikert C (2010). Genetic variation of the FADS1 FADS2 gene cluster and n-6 PUFA composition in erythrocyte membranes in the European Prospective Investigation into Cancer and Nutrition-Potsdam study. Br J Nutr.

