# Supplementary material for: Ethnicity, sex, FADS genetic variation, and hormonal contraceptive use influence delta-5- and delta-6-desaturase indices and plasma docosahexaenoic acid concentration in young Canadian adults: a cross-sectional study
Source: Nutr Metab (Lond). 2015 Apr 21;12:14. doi: 10.1186/s12986-015-0010-9 (PMC4410003; doi:10.1186/s12986-015-0010-9)
Supplement: Additional file 1: Table S1. — HWE values of FADS1 and FADS2 and their genotype frequencies. [file 12986_2015_10_MOESM1_ESM.pdf]

Additional table 1

HWE values of *FADS1* and *FADS2* and their genotype frequencies.

Gene name	SNPs	Caucasians						East Asians					
		Males			Females			Males			Females		
		HWE	Major allele	MAF	HWE	Major allele	MAF	HWE	Major allele	MAF	HWE	Major allele	MAF
FADS1	rs174547	0.66	T	0.34	0.48	T	0.34	0.89	C	0.44	8.8×10^{-6}	C	0.43
	rs412334	NP	G		0.48	G	0.15	NP	G		NP	G	
	rs695867	NP	A		NP	A		NP	A		NP	A	
	rs740006	NP	A		NP	A		NP	A		NP	A	
FADS2	rs174570	0.80	C	0.13	0.73	C	0.15	0.90	T	0.44	5.8×10^{-5}	T	0.43
	rs174576	0.80	C	0.37	0.73	C	0.35	0.90	A	0.43	5.8×10^{-5}	A	0.42
	rs174579	0.80	C	0.19	0.73	C	0.21	0.97	C	0.15	0.89	C	0.14
	rs174593	0.80	T	0.27	0.73	T	0.27	0.96	T	0.14	0.97	T	0.15
	rs174602	0.80	A	0.25	0.91	A	0.23	0.82	A	0.41	0.07	A	0.37
	rs174611	0.80	T	0.30	0.73	T	0.30	NP	T		NP	T	
	rs174626	0.80	T	0.49	0.73	T	0.50	0.82	T	0.33	0.97	T	0.33
	rs174627	0.80	C	0.17	0.70	C	0.15	NP	C		NP	C	
	rs17831757	0.80	T	0.11	0.73	T	0.13	NP	T		NP	T	
	rs2072114	0.80	A	0.17	0.73	A	0.14	0.90	A	0.43	0.07	A	0.43
	rs2845573	NP	T		0.51	T	0.09	0.82	T	0.42	0.07	T	0.43
	rs2851682	0.80	A	0.11	0.70	A	0.10	0.82	A	0.43	0.07	A	0.43
	rs482548	0.92	C	0.09	0.73	C	0.10	NP	C		NP	C	
	rs498793	0.39	G	0.40	0.51	G	0.41	0.82	G	0.10	0.52	G	0.09
	rs526126	0.80	C	0.20	0.33	C	0.19	0.78	C	0.17	0.97	C	0.19
	rs968567	0.80	G	0.16	0.73	G	0.17	NP	G		NP	G	

SNPs with HWE p-values less than 0.05 are not in Hardy-Weinberg Equilibrium. Caucasian Males: n = 113; Caucasian females: n = 298; East Asian Males: n = 98; East Asian Females: n = 277. Abbreviations: HWE, Hardy-Weinberg Equilibrium p-values; MA, minor allele; MAF, minor allele frequency; NP, not polymorphic (MAF = 0).
